# ^18^F-FDG PET/CT in Neurolymphomatosis: Report of 3 Cases

**Published:** 2014

**Authors:** Nguyen Xuan Canh, Ngo Van Tan, Tran Thanh Tung, Nguyen Truong Son, Simone Maurea

**Affiliations:** 1 Unit of PET/CT and Cyclotron, Choray Hospital, Hochiminh City, Vietnam; 2 Department of Hematology, Choray Hospital, Hochiminh City, Vietnam; 3 Dipartimento Di Scienze Biomediche Avanzate, Facoltá Di Medicina E Chirurgia, Università Degli Studi Di Napoli Federico II, Italia

**Keywords:** ^18^F-FDG, Nerve, Neurolymphomatosis, PET/CT, Plexus

## Abstract

Neurolymphomatosis is a rare manifestation of non-Hodgkin lymphoma characterized by infiltration of peripheral nerves, nerve roots, plexus and cranial nerves by malignant lymphocytes.

This report presents positron emission tomography/computed tomography (PET/CT)imaging with 2-deoxy-2-^18^F-fluoro-D-glucose (^18^F-FDG) in 3 cases of non-Hodgkin lymphoma with nerve infiltration, including one newly diagnosed lymphoma, one recurrent lymphoma in previous nerve lesions and one newly recurrent lymphoma. PET/CT could reveal the affected neural structures including cranial nerves, spinal nerve roots, brachial plexus, cervicothoracic ganglion, intercostal nerves, branches of the vagus nerve, lumbosacral plexus and sciatic nerves. There was relative concordance between PET/CT and MRI in detection of affected cranial nerves. PET/CT seemed to be better than MRI in detection of affected peripheral nerves.

^18^F-FDG PET/CT was a whole-body imaging technique with the ability to reveal the affected cranial nerves, peripheral nerves, nerve roots and plexus in non-Hodgkin lymphoma. A thorough understanding of disease and use of advanced imaging modalities will increasingly detect neurolymphomatosis.

## Introduction

Non-Hodgkin lymphoma (NHL) is one of the most common forms of hematologic cancers. The Age Standardized Rate (ASR) of NHL was 8.3 in males and 2.6 in females in Hanoi city and 2.8 in males and 2.1 per 100.000 in females in Hochiminh city, Vietnam(1). NHL can involve the central and peripheral nervous system. Neurolymphomatosis is a rare manifestation of NHL characterized by infiltration of peripheral nerves, nerve roots, plexus and cranial nerves by malignant lymphocytes.

Most commonly, neurolymphomatosis presents as a painful polyneuropathy or polyradiculopathy, followed by cranial neuropathy, painless polyneuropathy, and peripheral mononeuropathy. Diagnosis of neurolymphomatosis is challenging and requires integration of clinical information, imaging findings, and histopathologic examination of involved nerves or nonneural tissue and cerebrospinal fluid analysis (2).

Imaging modalities play an important role to reveal neurolymphomatosis. MRI findings may be enlargement and enhancement of affected cranial nerves, nerve roots, plexus and trunks of peripheral nerves.

Positron emission tomography/computed tomography (PET/CT) with 2-deoxy-2-^18^F-fluoro-D-glucose (^18^F-FDG) has been extensively used for staging and treatment monitoring of lymphoma (3, 4). PET/CT demonstrated high sensitivity for diagnosis of neurolymphomatosis allowing for evaluation of the affected nervous structures and the therapeutic responses to chemotherapy (5, 6).

From March 2009 to December 2013, we performed 818 cases of ^18^F-FDG PET/CT for lymphoma at Choray hospital, Vietnam and detected 3 cases of neurolymphomatosis from non-Hodgkin lymphoma. In this report, we described ^18^F-FDG PET/CT findings of these neurolymphomatosiscases.

## Case Report

All three patients were fasted for at least 6 hours with blood glucose level lower than 120 mg/dl before starting ^18^F-FDG PET/CT imaging. The patients were injected a dose of 5.18 MBq/kg (0.14 mCi/kg) of ^18^F-FDG. The whole-body and additional scans were performed at 60 minutes after ^18^F-FDG injection in a PET/CT scanner (Biograph True D w/true V, Siemens Medical System).

## Case report 1

A 53-year-old man developed mild pain in the left shoulder radiating down to arm with weakness and atrophy of the left arm from 1.5 year ago. He was diagnosed as inflammation of the brachial plexus and there was no improvement after treatment of high-dose prednisolone.

Ten months later, the clinical condition was getting worse and new symptoms and signs appeared including progressive atrophy of the left arm, ptosis and diplopia of the left eyelid and facial paresis. Neurological examination revealed palsy of the left cranial nerve III, VI, VII. There was no remission after treatment of immunoglobulin, azathioprine and corticoids.

In last two months, he has experienced weakness, unpleasant sensation of tingling and atrophy of the lower legs and urinary hesitancy. Neurological examination revealed palsy of the left cranial nerve III, IV, V, VI, VII, IX, X, XII. Cerebrospinal fluid analysis showed high protein content with no abnormal cells. EMG findings suggested the left brachial plexus neuropathy. Bone scintigraphy showed no skeletal abnormalities. Bone marrow examination had normal result.

**Figure 1:**
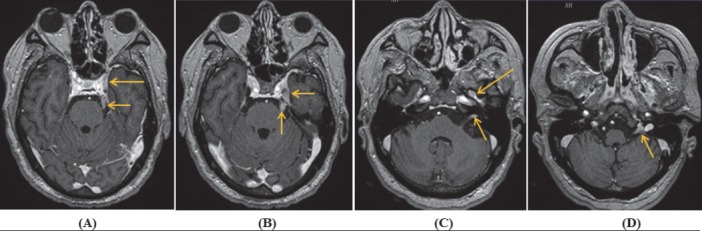
Axial contrast-enhanced T1W MRI showed enlargement and enhancement of the left cranial nerves: **(A)** complex of cranial nerves III, IV, VI, V1, V2 (long arrow); **(A, B)** cranial nerves V (short arrows); **(C)** cranial nerve V3 (long arrow) and complex of cranial nerves VII/VIII (short arrow); and **(D)** complex of cranial nerves IX, X, XI

**Figure 2:**
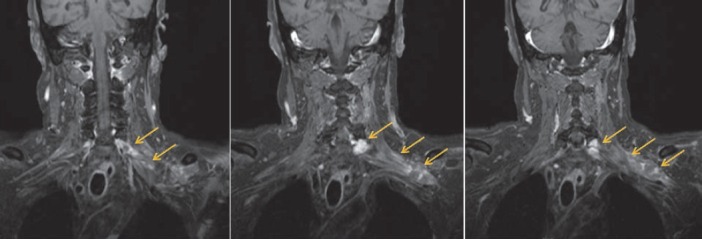
Coronal fat-suppressed, contrast-enhanced T1W MRI showed enlargement and enhancement of the left C7, C8 nerve roots and brachial plexus

Brain MRI revealed enhancement of the left cavernous sinus which was unable to clarify the involved cranial nerves III, IV, VI, V1 and V2. There were also enlargement and enhancement of the left cranial nerves V and V3, complex of cranial nerves VII/VIII and complex of cranial nerves IX, X, XI (Figure 1). Cervicothoracic MRI revealed enlargement and enhancement of the left C7, C8 nerve roots and brachial plexus (Figure 2). Lumbar MRI was non-contributory.

**Figure 3:**
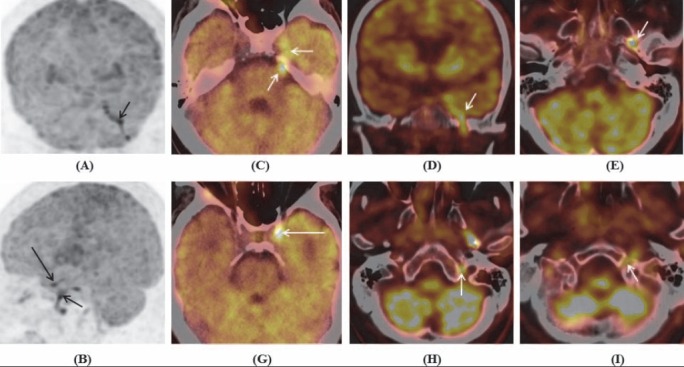
Maximum intensity projection of brain coronal **(A)** and sagital **(B)**, and PET/CT images of brain **(C-I)** showed increased FDG uptake in the left cranial nerves: **(A-E)** Cranial nerves V and V3; **(B, G)** complex of cranial nerves III, IV, VI, V1, V2 at cavernous sinus (long arrows); **(H)** complex of cranial nerves IX, X, XI at jugular foramen; and **(I)** cranial nerve XII at hypoglossal canal

**Figure 4:**
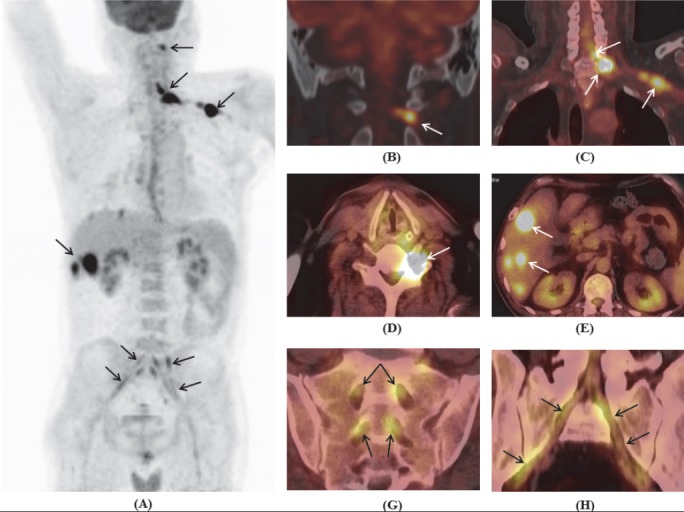
Maximum intensity projection of whole body **(A)** and PET/CT images **(B-H)** showed increased FDG uptake in the left C2 nerve root **(B)**; C5-C6 spinal cord, C7 nerve root, brachial plexus **(C,D)**; multiple focal lesions in liver **(E)**; and bilateral lumbosacral plexuses and along the sciatic nerves **(G,H)**

^18^F-FDG PET/CT imaging showed increased metabolism in the left cranial nerve V, foramen ovale (involving nerve V3), site of cavernous sinus (unable to clarify the involved nervesIII, IV, VI, V1, or V2), hypoglossal canal (involving nerve XII) and jugular foramen (unable to clarify the involved nerves IX, X or XI), the left C2 and C7 nerve roots, C5-C6 spinal cord and brachial plexus, bilateral lumbosacral plexuses, along the sciatic nerves and multiple focal lesions in liver (Figure 3,4).

Although the ultrasound detected impairment of the left brachial plexus, which was seemed difficult to perform an ultrasound-guided biopsy. Under the decision of clinicians, biopsy of liver lesion was done and the histopathology showed diffuse large B-cell lymphoma (DLBCL) with CD20 (+++). The patient refused the specific treatment and used herbal medicine and analgesic agents.

## Case report 2

A 36-year-old woman had a history of the right breast lump and pain radiating from the right side of neck to right hand associated with numbness from 2 years ago. The histopathology of breast lump showed diffuse large B-cell lymphoma (DLBCL) with CD20 (+++), Ki67 (+++), and negative CD3.

The diagnosis of neurolymphomato-sis involving the right brachial plexus was made by clinical symptoms and signs, MRI, PET/CT and EMG findings. She received a course of systemic chemotherapy comprising 6 cycles of R-CHOP (Rituximab, cyclophospha-mide, doxorubicin, vincristine, and predniso-lone) followed by radiation therapy. Complete response was evaluated by clinical features and PET/CT.

Six months after completion of the treatment, she complained of pain in her left arm. MRI and PET/CT showed abnormal findings of the left brachial plexus. Recurrence of lymphoma involving left brachial plexus was diagnosed and she was treated by 6 cycles of chemotherapy with R-ICE (Rituximab, ifosfamide, carboplatin, etoposide). Complete response was evaluated by clinical features and MRI.

**Figure 5:**
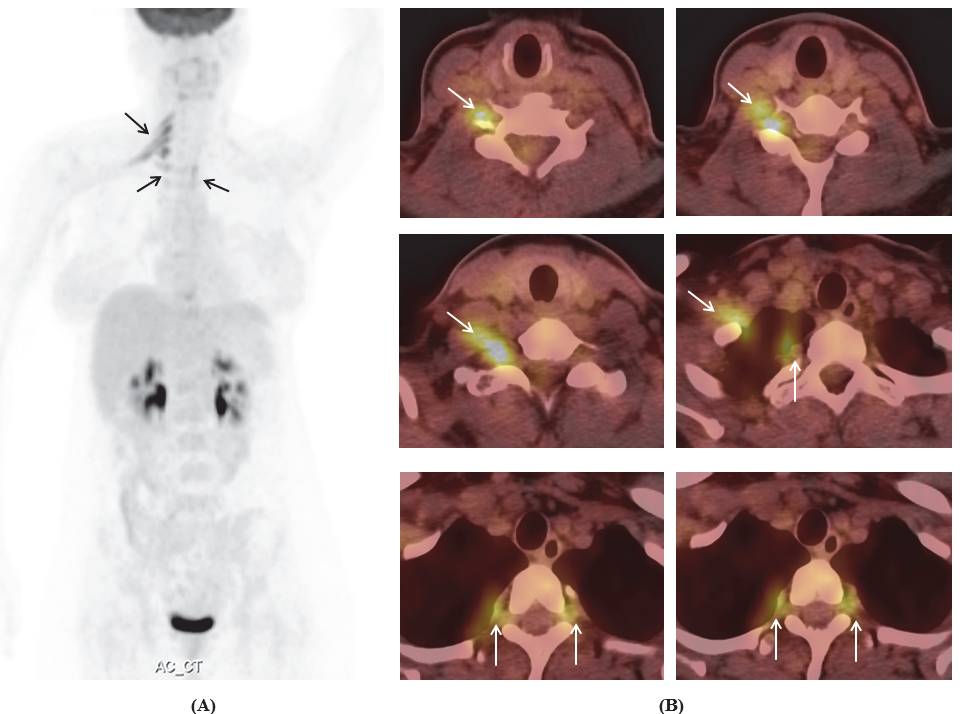
Maximum intensity projection of whole body **(A)** and PET/CT images **(B)** showed increased FDG uptake in the right C6, C7, C8, T1nerve roots and branchial plexus, and bilateral T2, T3 nerve roots

Six months later, she developed recurrent pain and numbness in bilateral shoulders and arms, more prominent in right-side, accompanied with ptosis of the right eyelid. Neurological examination revealed right III and IV cranial nerve palsy. Brain MRI did not show any abnormal finding in cranial nerve. ^18^F-FDG PET/CT imaging revealed increased metabolism in the right C6, C7, C8, T1 nerve roots and branchial plexus, and bilateral T2, T3 nerve roots (Figure 5).

She was diagnosed with recurrent neurolymphomatosis and treated by 6 cycles of chemotherapy with GEMOX regimen (gemcitabine and oxaliplatin). Clinical response was assessed.

## Case report 3

**Figure 6:**
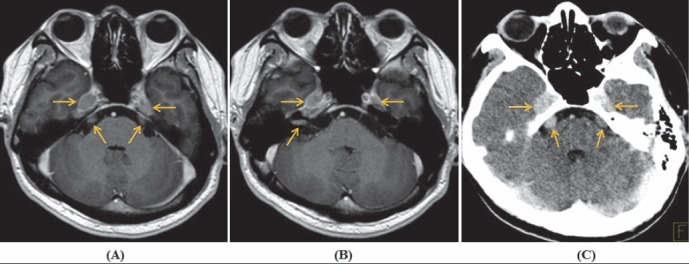
Axial contrast-enhanced T1W MRI **(A, B)** and CT image **(C)** showed enlargement and enhancement ofbilateral cranial nerve V and Gasser’s ganglions. Complex of the right cranial nerve VII/VIII was seen only on T1W image **(B)**

A 28-year-old man was diagnosed with T-cell lymphoma of neck lymph nodes and treated by 6 cycles of chemotherapy with CHOP regimen. Complete response was evaluated on clinical features and PET/CT. Three months later, he has facial pain. Brain MRI showed enlargement and enhancement of bilateral cranial nerve V and Gasser’s ganglions, and complex of the right cranial nerve VII/VIII (Figure 6).

**Figure 7:**
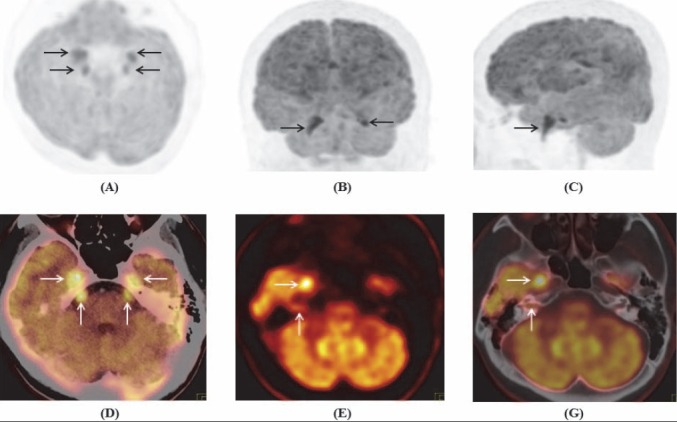
Maximum intensity projection of brain tranaxial **(A)** coronal **(B)** and sagital **(C)**; PET/CT **(D, G)** and PET images **(E)** showed increased FDG uptake in bilateral cranial nerve V, Gasser’s ganglions, and complex of the right cranial nerve VII/VIII

**Figure 8:**
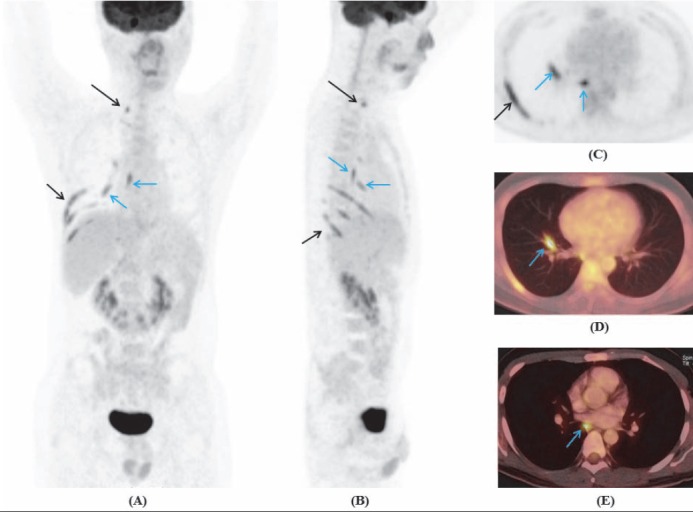
Maximum intensity projection of whole body coronal **(A)**, sagital **(B)**, thoracic transaxial **(C)**, and PET/CT images **(D, E)** showed increased FDG uptake of the right cervicothoracic ganglion (long arrow), along the 7^th^-10^th^ intercostal nerves, branches of the vagus nerve (blue arrows)

^18^F-FDG PET/CT with contrast-enhanced CT was performed with whole-body and brain scans, consecutively. CT images showed bilateral size-increased, contrast-enhanced cranial nerve V and Gasser’s ganglions (Figure 6). Brain PET/CT revealed hypermetabolic bilateral cranial nerve V and Gasser’s ganglions; and complex of the right cranial nerve VII/VIII (Figure 7). Whole-body PET/CT revealed hypermetabolic right cervicothoracic ganglion, along with 7^th^-10^th^ intercostal nerves and branches of the vagus nerve (Figure 8). The diagnosis was recurrent lymphoma with neurolymphomatosis.

## Discussion

We presented ^18^F-FDG PET/CT images in 3 cases of neurolymphomatosis from non-Hodgkin lymphoma. Neurolymphomatosis developed as initial manifestation or as recurrent disease. Nerve biopsy was not performed due to clinical circumstances. The combination of clinical history, physical examination, PET/CT, MRI and follow-up were used to confirm final diagnosis.

Neurolymphomatosis was primarily described as infiltration of the peripheral nervous system by lymphoma and non tumorous lymphocytes. Later, neurolymphoma-tosis has been reported to involve not only the peripheral nerves, but also cranial nerves. Majority of neurolymphomatosis were histopathologically confirmed as large B-cell lymphomas(7), and a small percentage as T-cell lymphomas(7-9). In the present report, there were 2 cases of neurolymphomatosis from diffuse large B-cell lymphoma and 1 from T-cell lymphoma.

There was relative concordance between PET/CT and MRI in detection of affected cranial nerves in neurolymphomatosis. Both two modalities successively identified multiple affected cranial nerves in 2 of 3 patients (patient 1 and 3). Because of the complexity of anatomy and normal course of some cranial nerves, both PET/CT and MRI vaguely demonstrated affected neural structures and could not differentiate the cranial nerves VII and VIII at the internal auditory canal (patient 3), the cranial nerves IX, X and XI at the jugular foramen and the cranial nerves III, IV, VI, V1 and V2 at the site of cavernous sinus (patient 1).

The cranial nerve XII was missed by MRI imaging and complex of cranial nerves VII/VIII was missed by PET/CT imaging in the first patient, who had neurological signs of involved cranial nerves. Moreover, PET/CT and MRI also failed to reveal any abnormal finding of cranial nerves in patient 2 with a palsy of the right III and IV cranial nerves, which was clinically improved after 6 cycles of chemotherapy. These findings suggest that PET/CT and MRI were not completely sensitive to identify impaired cranial nerves and neurological signs and symptoms of impaired cranial nerves could appear earlier than imaging findings.

The present report demonstrats that ^18^F-FDG PET/CT was able to reveal neurolympho-matosis involving spinal nerve roots, brachial plexus, lumbosacral plexus, intercostal nerves, cervicothoracic ganglion and branches of the vagus nerve, while MRI only reveals abnormal findings of cervicothoracic nerve roots and brachial plexus in patient 1 and 2 and was not contributory for diagnosis of lumbosacral plexus involvement in patient 1. MRI was not performed to evaluate the affected cervicothoracic ganglion, intercostal nerves and branches of the vagus nerve in patient 3.

Imaging studies have been proved to be powerful in diagnosis of neurolymphomatosis. The International Primary CNS Lymphoma Collaborative Group retrospectively analyzed and showed that PET and PET/CT may be more sensitive than MRI in diagnosis of neuro-lymphomatosis. PET and PET/CT presented abnormal findings of neurolymphomatosis in 16/19 patients (84%), compared with 36/47 patients (77%) in MRI (5). Many single case reports also demonstrated the high value of ^18^F-FDG PET/CT in establishing the diagnosis of NHL with neurolymphomatosis (10-19). There was a similarity between the present report and the other case reports on the capacity of ^18^F-FDG PET/CT in identifying impairments of the cranial nerve V and V3 (10, 11), the cranial nerve complex VII/VIII (10), the cranial nerve X (12, 13), the brachial plexus (13-17), the lumbosacral nerve root and plexus(16, 17), and the sciatic nerves (13, 16, 18, 19). Similar to reports of Choi YJ et al. (15) and Lin M et al. (16), the present report found that ^18^F-FDG PET/CT and MRI both were quite good to detect the brachial plexus, while the case report of Salm LP et al. presented ^18^F-FDG PET/CT findings of the brachial plexus suggesting neurolymphoma-tosis, which was not identified on MRI of the brachial plexus(14). Additionally, ^18^F-FDG PET/CT was better than MRI to detect lumbosacral plexus impairment in the present report and it is similar to the case reports of Lin M et al. (16) and Kajáry K et al.(19).

MRI may have the potential to detect the affected cranial nerves, but it is not always optimal to visualize the peripheral nerves involved in neurolymphomatosis. In the other hand, MRI findings were not specific for neurolymphomatosis and might sometimes be seen in acute or chronic inflammatory radiculoneuropathies, in inflammatory pseudotumor, and in malignant tumors of the peripheral nerve sheath (5). ^18^F-FDG PET/CT was sensitive in detecting abnormal hypermetabolism of cranial and peripheral nerves; however, these findings could be seen not only in non-Hodgkin lymphoma, but also in leukemia with neurolymphomatosis (20). Therefore, combination of multiple modalities including clinical and imaging findings in patients with known lymphoma may be essential to diagnose neurolymphomatosis.

The present report had certain limitation. A whole-body MRI has not been performed to compare side-by-side with ^18^F-FDG PET/CT and nerve biopsy has not been done for validation.

In conclusion, 18F-FDG PET/CT was a whole-body imaging technique with the ability to reveal the affected cranial nerves, peripheral nerves, nerve roots and plexus in non-Hodgkin lymphoma. A thorough understanding of disease and use of advanced imaging modalities will increasingly detect neurolymphomatosis.
